# SARS-CoV-2 antibody seroprevalence in Togo: a national cross-sectional household survey, May–June, 2021

**DOI:** 10.1186/s12889-022-14794-2

**Published:** 2022-12-08

**Authors:** Yao Rodion Konu, Siaka Condé, Fifonsi Gbeasor-Komlanvi, Arnold Junior Sadio, Martin Kouame Tchankoni, Joel Anani, Alexandra Bitty-Anderson, Bisimwa Ruhana Mirindi, Fatoumata Binta Tidiane Diallo, Moustapha MIjiyawa, Anoumou Claver Dagnra, Didier Koumavi Ekouevi

**Affiliations:** 1 Bordeaux Population Health Centre, UMR 1219, University of Bordeaux, National Institute for Health and Medical Research (INSERM), Research Institute for Sustainable Development (IRD), Bordeaux, France; 2 Département de Santé Publique, Université de Lomé, Lomé, Togo; 3 Centre de Recherche et de Formation en Santé Publique (CFRSP), Université de Lomé, Lomé, Togo; 4 Centre Africain de Recherche en Epidémiologie et en Santé Publique (CARESP), Lomé, Togo; 5 Togo Office, World Health Organization (WHO), Lomé, Togo; 6 Ministère de la Santé, de l’Hygiène Publique et de l’accès universel aux soins, Lomé, Togo; 7 Laboratoire de Biologie Moléculaire et d’Immunologie (BIOLIM), Université de Lomé, Lomé, Togo

**Keywords:** Seroprevalence, SARS-CoV-2, General population, Togo

## Abstract

**Background:**

The extent of SARS-CoV-2 circulation in African countries is still unclear. Seroprevalence studies are a common approach to epidemiological surveillance, allowing estimation of the proportion of people who have had contact with the virus. We aimed at estimating the seroprevalence of anti-SARS-CoV-2 antibodies and associated factors in Togo at the national level in 2021 according to age groups, gender, and place of residence (rural or urban).

**Methods:**

From 15 May to 31 June 2021, we conducted a nationally representative cross-sectional serological survey in 12 health districts (two districts per health region) in the > 5 years old population in Togo. The Wantai SARS-CoV-2 total antibody assay S protein receptor-binding domain-based ELISA (Wantai Biological Pharmacy Enterprise Co.; Beijing, China) was used to determine the presence of SARS-CoV-2 total antibodies in plasma. Crude and weighted seroprevalences (weighted by age, sex and place of residence) were calculated and then weighted seroprevalences were adjusted according to sensitivity and specificity of the ELISA test. Finally, logistic regression models were performed in order to describe factors associated.

**Results:**

Of the 7593 participants, the overall weighted and adjusted seroprevalence of total anti-SARS-CoV-2 antibodies was 65.5% (CI95%: 18.9–21.1). Urban dwellers, young adults (30–49 years) and vaccinated individuals were significantly more likely to be seropositive.

**Conclusion:**

The high seroprevalence we observed is consistent with observations across West Africa. Quantification of the level of immunity in the population is needed to know how close we are to herd immunity. In the meantime, vaccination against the COVID-19 remains necessary.

## Background

Since its onset in December 2019 in China, the COVID-19 pandemic continues to spread around the world. As of April 25th, 2022, the World Health Organization (WHO) reported over 510 million cumulative cases for 6 million deaths worldwide [1]. The African continent, whose population represents 17% of the world’s population, recorded only 1.7% of the COVID-19 cases in the world and 2.9% of the deaths. Indeed, only 8.7 million confirmed cases and 171,000 deaths were documented in 47 countries of the WHO African Region [1].

Early on, it became apparent that surveillance data underestimated the burden of the COVID-19 pandemic [2], particularly in sub-Saharan African countries which have limited capacity for contact tracing, diagnostic testing, and surveillance [3]. So, the COVID-19 detected cases are mainly limited to symptomatic persons, contacts of confirmed cases, and travelers. Therefore, the prevalence of SARS-CoV-2 infection remains poorly known worldwide and in sub-Saharan Africa in particular [4, 5].

Seroprevalence studies are a common approach to estimate the proportion of individuals previously infected with the virus in a given population [6]. They are essential for understanding the true extent of infection overall, by demographic group, and by geographic area [4]. Because anti-SARS-CoV-2 antibodies are predictive of immune protection [7], seroprevalence studies also provide information on population protection levels. They are therefore important for scenario modeling, public health planning and national policies in response to the pandemic [4]. In Europe, America and Asia, a number of population-based seroprevalence studies have been conducted from 2020 onwards [4, 5, 8]. With technical and financial support from WHO, similar studies have been conducted in resource-limited countries using a standardized protocol [4, 9].

In Togo, the first case of COVID-19 was reported on March 6th, 2020. As of April 25th, 2022, Togo has experienced three epidemic waves (April 2021, August 2021 and January 2022) with a total of 36,979 confirmed cases and 273 deaths [1]. In order to document the magnitude of the Covid-19 pandemic, a first seroprevalence study was conducted before the first epidemic wave, 2 months after the report of the first cases in Togo (April–May 2020) among populations with at high risk of SARS-CoV-2 infection. That first study reported a seroprevalence of 0.8% [10]. We report in this article the results of a national seroprevalence survey conducted in May 2021 after the first epidemic wave.

The primary objective of this study was to estimate the seroprevalence of anti-SARS-CoV-2 antibodies and associated factors in Togo at the national level in 2021 according to age groups, gender, and place of residence (rural or urban).

## Methods

### Study design and period

A nationally representative descriptive and analytic cross-sectional household study was conducted between May 5th and June 30th, 2021, after the first epidemic wave in Togo (April–May 2021).

### Setting

Togo is a West African country with a surface area of 56,600 km^2^ and a population of 7.8 million inhabitants in 2020 [11]. It is a country with a gross domestic product of $1593 per capita in 2018. Its human development index is constantly increasing and went from 0.405 in 1990 to 0.513 in 2018, which ranked it 167th in the world [12]. Regarding the health system, the country is divided into six health regions composed with 39 health districts.

### Study population

The target population was inhabitants of the six health regions of Togo, whether they lived in urban, peri-urban or rural areas, recruited in 12 health districts (two districts per health region). Subjects were included if they met the following criteria: i) they were 5 years of age or older, ii) they resided in a randomly selected household, iii) they gave free and informed consent to participate in the study and to provide a blood sample. For minors under the age of 18, in addition to the subject’s assent, consent from the legal guardian was required.

### Sample size

For the sample size calculation at a health region level, we used a modified single proportion formula with the design effect as recommended by the Standardized Monitoring and Assessment of Relief and Transitions (SMART) methodology [13]. The design effect also known as “cluster effect” is a correction factor that takes into account the heterogeneity of clusters with respect to the indicator being measured. According to this, considering an expected seroprevalence of 4%, precision of 1%, design effect of 2 and an estimated 10% of nonresponse rate we estimated a total of 1229 subject per health region thus 7374 for the whole country. The estimate of the sample size by region was finally adjusted based on the proportion of the population size of each health region.

### Subject selection

Participants were selected by a multistage cluster sampling. i) In the first stage, in each health region, two health districts were selected according to the incidence (number of cases of the COVID-19 reported in each region) based on the national data of the COVID-19 surveillance as of March 1st, 2021. Thus, the district with the highest and lowest incidence was included according to the WHO protocol for age-stratified population-based sero-epidemiological surveys for the COVID-19 infection [14]. ii) At the second level, in each district, a stratification was carried out according to the place of residence (urban or rural). In each setting, a random draw of 10 neighborhoods was then conducted. iii) At the third level, in each neighborhood, a systematic draw of 20 households was conducted. Each household constituted a cluster. Finally, in each household, a maximum of 3 individuals > 5 years were randomly selected using a random number table, plus the chief of the household was systematically included.

### Data collection

Data were collected during a face-to-face interview at the household using a digitalized questionnaire providing information on: sociodemographic characteristics, epidemiological link, knowledge, attitudes and practices regarding the COVID-19, history of the COVID-19 symptoms and vaccination status regarding the COVID-19.

In addition to the collected data, a 4 ml peripheral venous blood sample was taken from each participant in a tube containing ethylene diamine tetra acetic acid (EDTA) anticoagulant at the district health center. The whole blood samples were kept in a container at + 4 °C and then transferred to the district laboratory within 3 hours for preparation of plasma aliquots. The aliquots were frozen at − 25 °C and transported to the national virology laboratory for quality control and antibody testing.

Data collection was carried out at participant’s home by qualified research personnel trained in the survey procedures by using a mobile application dedicated to this survey. Blood samples were collected by nurses from district hospitals.

### Biological analyses

Qualitative tests for anti-SARS-CoV-2 antibodies were performed by enzyme-linked immunosorbent assay (ELISA) in the reference laboratory for the control of cis-viral infections at the Sylvanus Olympio University Hospital in Lomé. Plasma samples were kept frozen at − 25 °C until analysis. To determine the presence of SARS-CoV-2 total antibodies in plasma, the Wantai SARS-CoV-2 total antibody assay S protein receptor-binding domain-based ELISA (Wantai Biological Pharmacy Enterprise Co.; Beijing, China) was used. This test is intended to be used to identify individuals with an adaptive immune response to SARS-CoV-2, indicating recent or previous infection [15]. According to the manufacturer, the sensitivity and specificity of the test are 94.36 and 100%, respectively [15].

### Statistical analysis

Results were presented as frequencies and proportions for qualitative variables. Quantitative variables were presented as median with their interquartile range (IQR). The overall and district seroprevalence of anti-SARS-CoV-2 antibodies were estimated with their 95% confidence interval (95% CI). Crude and weighted seroprevalences (weighted by age, sex and place of residence) were calculated and then weighted seroprevalences were adjusted according to the sensitivity and specificity of the ELISA test.

Weighted seroprevalence estimates were computed using the “survey” package (*svydesign* and *svyciprop* functions) of R version 4.1.3. The Rogan-Gladen formula was then used to adjust seroprevalence estimates to account for the sensitivity and specificity of the ELISA test [16].

Regression analyses were performed to assess factors associated with SARS-CoV-2 infection according to the ELISA test. Multivariable analyses by logistic regression models with household random intercepts to account for within-household clustering were performed. A Wald Chi-square test was carried out on each univariable model, and all factors that had a *p*-value < 0.20 were entered into the multivariable analysis. The full multivariable models were subsequently finalized using a stepwise, backward elimination approach (*p*-value < 0.05). Individuals with missing covariables were not included in the regression analysis.

### Ethical considerations

The study was approved by the Bioethics Committee for Health Research (CBRS) of the Ministry of Health, Public Hygiene and Universal Access to Health Care (No. 002/2021/CBRS of February 09, 2021). Written informed consent was obtained prior to any administration of the questionnaire and any sampling of the participants. For subjects ages 5 to 17 years, assent was obtained in addition to the written consent of the parent or legal guardian prior to any data collection and blood samples.

### Guidelines

The present study aligns with the World Health Organization UNITY standardized protocols for international investigation during the COVID-19 pandemic [9].

## Results

### Socio-demographic characteristics

A total of 7593 people out of the 8066 approached in 1956 Togolese households were included in the analyses. Included participants were aged 6 to 99 years old with a median of 32 years old (interquartile range, IQR = 17–44). The majority (54.8%) of participants were female. More than half of the sample was recruited in urban areas, (*n* = 4000; 52.7%). Regarding the level of education, 19.1% of the participants had never attended school, while 7.1% had a university degree. Of all the adult participants eligible to the COVID-19 vaccination, 21.1% (1316/6301) were vaccinated at the time of the study. Table 1 presents the sociodemographic characteristics of the participants.

**Table 1:** Socio-demographic characteristics and vaccination history of participants, March–June 2021 (*N* = 7593)

	Grand Lomé	Maritime	Plateaux	Centrale	Kara	Savanes	Total	P^*^
	***N*** = 1938	***N*** = 1229	***N*** = 1521	***N*** = 799	***N*** = 1019	***N*** = 1087	***N*** = 7593	
**Age (years), median [IQR]**	35 (23–49)	32 (13–45)	28 (12–40)	30 (17–47)	31 (19–46)	32 (10–40)	32 (17–44)	**< 0,001**
**Age groups (years)**								**< 0,001**
< 10	100 (5.2)	184 (15.0)	223 (14.6)	113 (14.1)	99 (9.7)	218 (20.1)	937 (12.3)	
10–14	115 (5.8)	136 (11.1)	230 (15.1)	51 (6.4)	82 (8.0)	124 (11.4)	738 (9.7)	
15–19	126 (6.5)	77 (6.3)	124 (8.2)	58 (7.3)	84 (8.2)	14 (1.3)	483 (6.4)	
20–29	411 (21.2)	167 (13.6)	222 (14.6)	158 (19.8)	183 (18.0)	125 (11.5)	1266 (16.6)	
30–39	430 (22.2)	217 (17.7)	313 (20.6)	133 (16.6)	217 (21.3)	330 (30.3)	1640 (21.6)	
40–49	290 (15.0)	214 (17.4)	214 (14.1)	108 (13.5)	142 (13.8)	185 (17.0)	1153 (15.2)	
50–59	255 (13.2)	137 (11.1)	109 (7.2)	92 (11.5)	108 (10.6)	66 (6.1)	767 (10.1)	
60–70	145 (7.5)	64 (5.2)	43 (2.8)	53 (6.5)	61 (6.0)	16 (1.5)	382 (5.0)	
70+	66 (3.4)	33 (2.7)	43 (2.8)	33 (4.1)	43 (4.2)	9 (0.8)	227 (3.0)	
**Sex**								0.106
Female	1052 (54.3)	688 (56.0)	767 (50.4)	469 (58.7)	638 (62.6)	550 (50.6)	4164 (54.8)	
Male	886 (45.7)	541 (44.0)	754 (49.6)	330 (41.3)	381 (37.4)	537 (49.4)	3429 (45.2)	
**Location**								**< 0.001**
Rural	173 (8.9)	729 (59.3)	966 (63.5)	492 (61.6)	579 (56.8)	654 (60.2)	3593 (47.3)	
Urban	1765 (91.1)	500 (40.7)	555 (36.5)	307 (38.4)	440 (43.2)	433 (39.8)	4000 (52.7)	
**Education level**								**< 0.001**
No formal instruction	224 (11.5)	145 (11.8)	416 (27.4)	166 (20.8)	240 (23.6)	300 (27.6)	1491 (19.6)	
Primary	666 (34.4)	557 (45.3)	706 (46.4)	305 (38.2)	325 (31.9)	591 (54.4)	3150 (41.5)	
Secondary	750 (38.7)	444 (36.1)	378 (24.9)	302 (37.8)	356 (34.9)	168 (15.5)	2398 (31.6)	
University	298 (15.4)	83 (6.8)	21 (1.3)	26 (3.2)	98 (9,6)	28 (2.5)	554 (7.3)	
**Already vaccinated against COVID-19 (*n*** **= 6301)**								**< 0.001**
No	1259 (68.6)	842 (86.9)	1028 (85.8)	492 (74.4)	746 (84.5)	602 (79.6)	4969 (78.9)	
Yes	575 (31.4)	127 (13.1)	170 (14.2)	169 (25.6)	137 (15.5)	154 (20.4)	1332 (21.1)	

*IQR* interquartile range

^*^Chi-squared test with Rao & Scott’s second-order correction; Design-based Kruskal Wallis test

### Seroprevalence

Overall, the seroprevalence weighted by age, sex and residence (rural/urban) and adjusted for intrinsic test characteristics was 65.5% (95% confidence interval, 95%CI: 64.3–66.6) (Table 2). Seroprevalence was 66.2% (64.6–67.8) in men. The 30–39 and 40–49 age groups had the highest weighted and adjusted seroprevalences with 74.0% (70.8–77.3) and 71.8% (67.7–75.8) of positive serology, respectively. The highest seroprevalences of markers of SARS-CoV-2 infection were recorded in urban areas (71.3% [69.5–73.1] vs 62.0% [60.6–63.5]). Seroprevalence of markers of SARS-CoV-2 infection was significantly higher in participants who received at least one dose of the COVID-19 vaccine. Indeed, the adjusted seroprevalence of antibodies to SARS-CoV-2 was 86.5% (84.2–88.8) in vaccinated participants and 65.1% (63.7–67.4) in unvaccinated participants.

**Table 2:** Seroprevalence of SARS-CoV-2 infection by age, sex, area of residence (location) and vaccination status, March – June 2021 (*N* = 7593)

			Seroprevalence (95% confidence interval)		
	N	n	Crude % [95%CI]	Age-sex-location-weighted^**a**^ % [95%CI]	Age-sex-location-weighted. Adjusted by test characteristic^**b**^ % [95%CI]
**Overall**	7593	4884	64.3 [63.2–65.4]	61.8 [60.7–62.9]	65.5 [64.3–66.6]
**Age group (years)**					
< 10	937	445	47.5 [44.3–50.7]	47.2 [44–50.4]	50 [46.6–53.4]
10–14	738	424	57.5 [53.9–61]	56 [52.8–59.2]	59.3 [55.9–62.7]
15–19	483	321	66.5 [62.2–70.7]	66.1 [62.6–69.5]	70 [66.3–73.7]
20–29	1266	830	65.6 [62.9–68.2]	63.4 [60.6–66.2]	67.2 [64.2–70.2]
30–39	1640	1145	69.8 [67.6–72]	69.8 [66.8–72.9]	74 [70.8–77.3]
40–49	1153	789	68.4 [65.7–71.1]	67.7 [63.9–71.5]	71.8 [67.7–75.8]
50–59	767	518	67.5 [64.2–70.8]	65.7 [60.5–70.9]	69.7 [64.2–75.2]
60–70	382	270	70.7 [66.1–75.2]	66.9 [64–69.8]	70.9 [67.8–74]
70+	227	142	62.6 [56.3–68.9]	60.1 [57.5–62.7]	63.7 [61–66.4]
**Sex**					
Female	4164	2689	64.6 [63.1–66]	61.1 [59.5–62.6]	64.7 [63.1–66.3]
Male	3429	2195	64 [62.4–65.6]	62.5 [61–64]	66.2 [64.6–67.8]
**Location**					
Rural	3593	2184	60.8 [59.2–62.4]	58.5 [57.2–59.9]	62.0 [60.6–63.5]
Urban	4000	2700	67.5 [66.0–69.0]	67.3 [65.6–69]	71.3 [69.5–73.1]
**Already vaccinated against COVID-19 (*n*** **= 6240)**					
No	4924	3102	63.0 [61.6–64.3]	61.6 [59.1–63.0]	65.1 [63.7–67.4]
Yes	1316	1102	83.7 [81.7–85.7]	81.7 [79.5–83.8]	86.5 [84.2–88.8]

N: Sample size

n: SARS-COV-2 positive subjects

^a^Weighted on age, gender and location

^b^Weighted and then adjusted according to the characteristics of the test

### Seroprevalence by health area

**Fig. 1:**
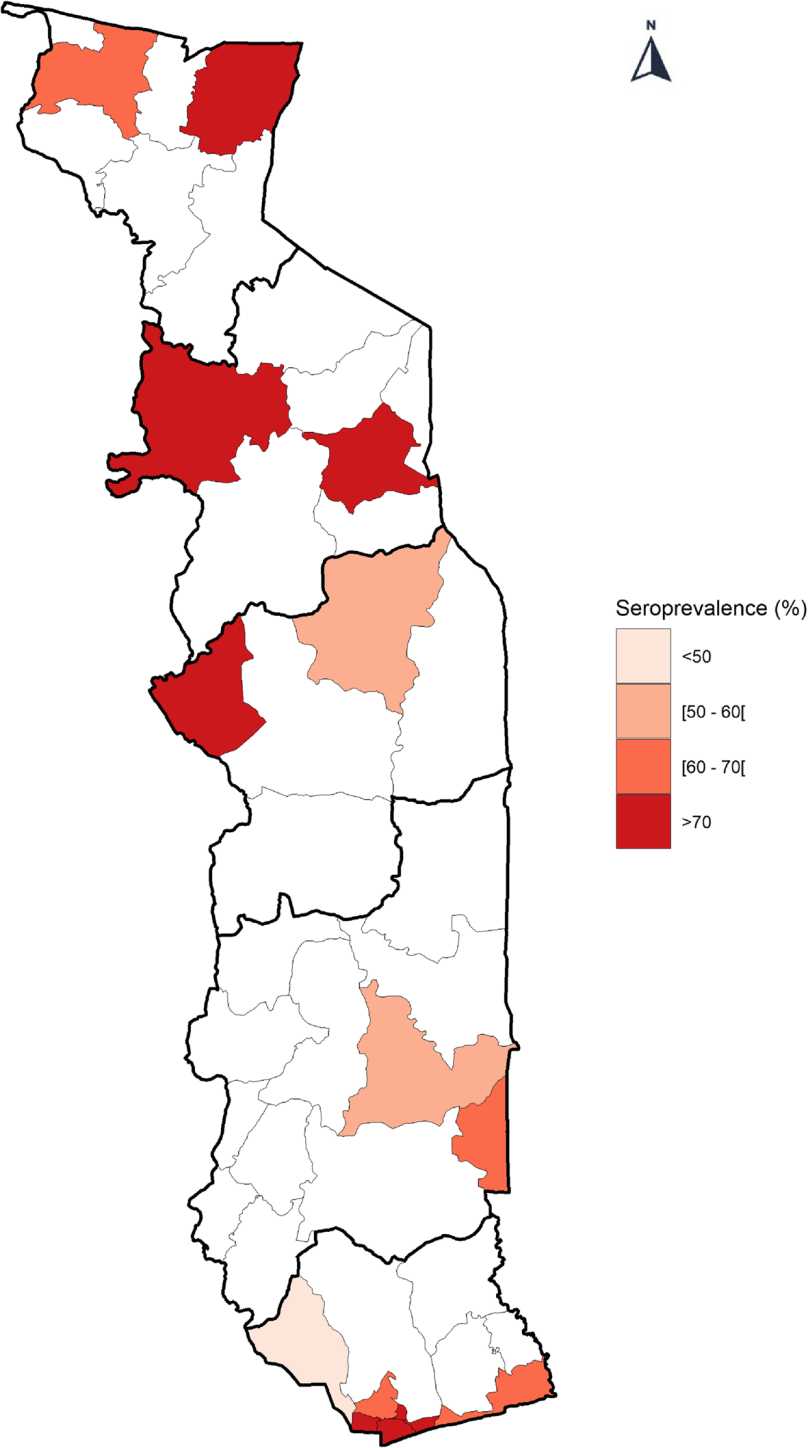
Seroprevalence by district, Sero-CoV survey, May–June 2021

The health regions of Grand Lomé and Kara reported the highest levels of seroprevalence, 75.5 and 73.2% respectively. Some districts initially designated as low incidence districts ended up recording higher prevalence and vice versa. In fact, in the Central Region, where surveillance data indicated that Tchaoudjo was a high-incidence district and Mo a low-incidence district, seroprevalence was 59.9% (56.4–63.3) in Tchaoudjo compared to 82.0% (74.3–89.7) in the Mo district (Fig. 1).

### Factors associated with the presence of anti-SARS-CoV-2 antibodies

**Fig. 2:**
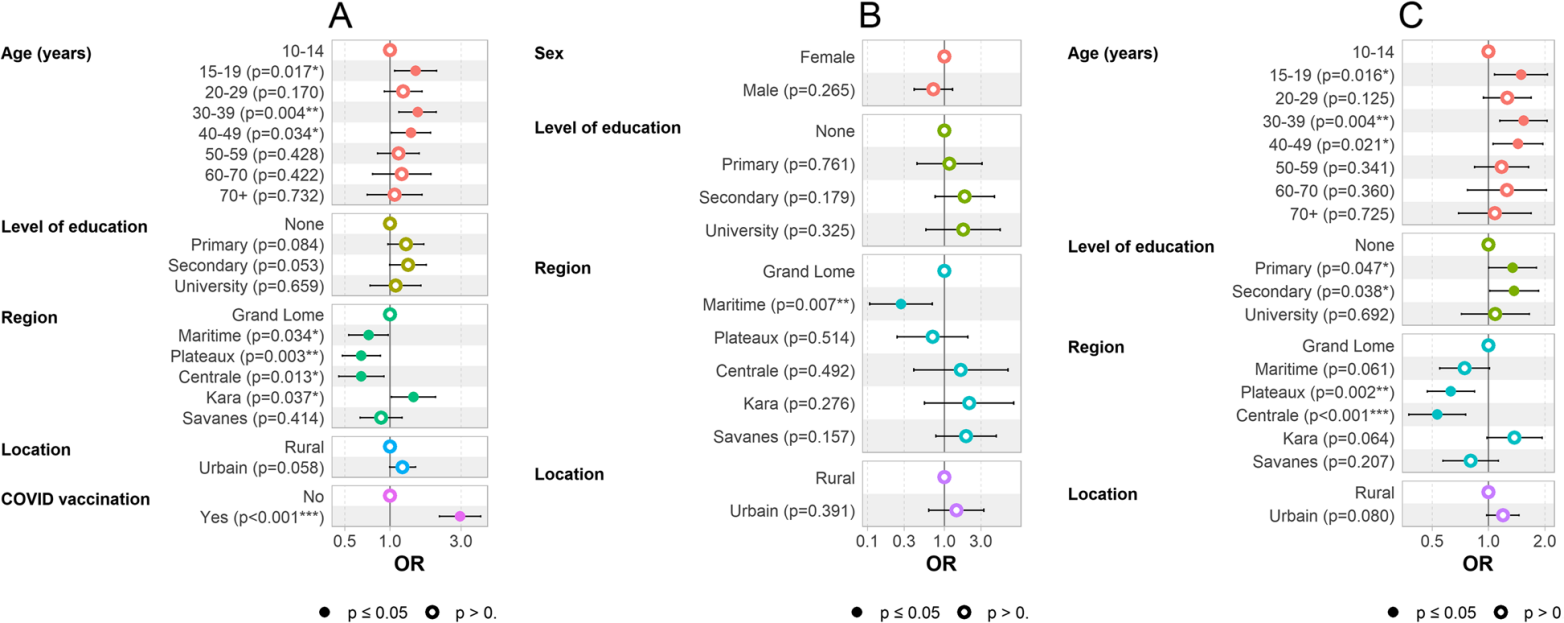
Factors associated with the presence of anti-SARS-CoV-2 antibodies, final multivariate models, Sero-CoV Togo survey, March – June 2021 (**A**- whole sample (*N* = 7593), **B**-vaccinated subjects (*N* = 1316), **C**- unvaccinated subjects (*N* = 4924)). *The final multivariable model for **B** is better adjusted without the age variable

The binary logistic regression model performed on the entire sample is presented in Fig. 2A. Overall, age (*p* = 0.011), health region (*p* < 0.001) and the COVID-19 vaccination status (*p* < 0.001) were associated with the presence of SARS-CoV-2 antibodies. This analysis revealed that participants 15–19 (AOR = 1.48; IC95% = 1.07–2.05; *p* = 0.017), 30–39 (AOR = 1.53; IC95% = 1.15–2.04; *p* = 0.004) and 40–49 (AOR = 1.39; IC95% = 1.02–1.87; *p* = 0.034) years of age had a significantly higher risk of being SARS-CoV-2 antibodies positive compared to 10–14 year olds.

The binary logistic regression analysis in the group of vaccinated subjects is presented in Fig. 2B and describes the factors associated with the production of anti-SARS-CoV-2 antibodies in vaccinated subjects. It can be seen that vaccinated subjects from the Maritime region were 73% less likely (AOR = 0.27, IC95% = 0.11–0.70; *p* = 0.007) to produce antibodies to SARS-CoV-2 than subjects from Grand Lomé.

Figure 2C shows the factors associated with SARS-CoV-2 antibodies presence in unvaccinated individuals. Overall, age (*p* = 0.029) and health region (*p* < 0.001) were significantly associated with the SARS-CoV-2 antibodies. The seroprevalence of SARS-CoV-2 in 15–19, 30–39 and 40–49 year olds was significantly higher than in 10–14 year olds. According to the same data, living in the Plateaux and Central regions was a factor that significantly reduced the risk of being SARS-CoV-2 antibody positive (AOR = 0.63; 95%CI = 0.47–0.84; *p* = 0.002 and AOR = 0.53; 95%CI = 0.38–0.76; *p* < 0.001).

## Discussion

To our knowledge, this is the only national seroprevalence study conducted in the context of the COVID-19 pandemic in Togo. The present study was conducted between the first and second epidemic waves and shows that nearly two thirds of subjects aged 5 years and older (65.5%) acquired SARS-CoV-2 antibodies between March 2020 and June 2021 in Togo, with a preponderance in urban areas and among adult subjects (30 to 49 years). This prevalence confirms a high circulation of SARS-CoV-2 in the general population of Togo in 2021.

Comparison of SARS-CoV-2 seroprevalence studies is very difficult and should be done with caution. Indeed, the results of these studies are influenced by the study population, the period (before, after an epidemic wave) and the characteristics of the tests. In Togo, a first study conducted in May and June 2020 in Lomé among populations at high risk of infection reported a seroprevalence of 0.9% [10]. Although these results may be underestimated, the observed difference reflects the spread of the virus in the population 1 year and after the first epidemic wave in Togo.

Population-based seroprevalence studies similar to ours, following the WHO generic protocol and using the same ELISA tests, have been performed in the West African sub-region. The reported seroprevalence were 67.9% in Ghana [17] and 78.9% in Nigeria [18]. In both countries, youth (15–19 years) and young adults (30–39 years) were most affected by the infection [17, 18]. These proportions corroborate those observed in Togo.

A meta-analysis performed on data published globally between January 1st and December 31st, 2020, also confirms the high circulation of the virus in sub-Saharan Africa. Median seroprevalence varied by continent, from 0.6% in Southeast Asia, East Asia and Oceania to 19.5% in sub-Saharan Africa (*p* < 0.001) [5]. A second meta-analysis [19], taking into account data from Africa covering the period from January 2020 to December 2021 reported 151 seroprevalence studies on the continent. SARS-CoV-2 seroprevalence ranged between 3.0% in the second quarter of 2020 to 65.1% in the third quarter of 2021 [19]. Seroprevalence was highly heterogeneous within countries. It was lower in rural geographic areas as demonstrated in our study [19]. A final meta-analysis of individual data from 27,735 participants collected between April 2020 and April 2021 in Africa reported a seroprevalence of anti-SARS-CoV-2 antibodies of 22% with very high heterogeneity [20]. In summary, studies in Africa report a high level of SARS-CoV-2 circulation based on the presence of IgG. However, a cross reaction was reported. Indeed, several studies suggest that Individuals with acute malaria infection or living in malaria endemic settings generated high levels of antibodies that could substantially cross-react with the SARS-CoV-2 serological assays [21, 22]. In a study conducted by the team of Dorkenoo et al., among a population of febrile subjects during the pandemic in Lomé, a higher presence of anti-SARS-CoV-2 antibodies (IgG) was demonstrated in patients diagnosed with malaria [23].

In order to have better idea of the circulation of the virus during this long-lasting pandemic, it would have been more relevant to conduct seroprevalence studies at regular intervals. This approach has been adopted in some countries in the sub-region, notably in Guinea, where a study reports three successive seroprevalence surveys, 3 months apart, using multistage cluster sampling to measure the extent and dynamics of the SARS-CoV-2 epidemic in Conakry, the capital city [24]. Seroprevalence increased from 17.3% in December 2020 in the first survey to 28.9% in March/April 2021 and to 42.4% in June 2021. This significant overall trend of increasing seroprevalence (*p* < 0.0001) was also significant in each age group. Such an increase could be explained by a high circulation of the virus in the community or by the introduction of the SARS-CoV-2 vaccine in the second quarter of 2021. Indeed, in this study, it has been reported that among seropositive participants in the last study 18% were vaccinated vs 0% in the first study [24] illustrating sustained transmission within the community at large. These data may contribute to the development of efficient response strategies.

Overall, one fifth of our sample was vaccinated. This proportion is high considering the study period. A social desirability bias cannot be excluded. However, the seroprevalence of anti-SARS-CoV-2 antibodies was higher in vaccinated subjects of all ages. The presence of antibodies remains a qualitative marker that should be coupled with the titration of neutralizing antibodies in order to demonstrate the level of immunization and to guide vaccination policy. This issue should be discussed in a context of strong hesitation with the COVID-19 vaccine coverage remaining below 38% in April 2022 in Togo. Similarly, the issues around vaccine boost doses deserve particular attention.

Given the variation in antibody kinetics over time, it is important to repeat seroprevalence surveys and couple them with antibody titration. This would document the long-term persistence of anti-SARS-CoV-2 antibodies that may protect against reinfection. Indeed, there is evidence that by significantly increasing neutralizing antibody titers, a single dose vaccination enhances protection against variants. Assessment of anti-SARS-CoV-2 antibody kinetics is essential to predict protection against reinfection and durability of vaccine protection [25]. Based on our results, Togo might be on its way to achieving herd immunity, if the virus continues to circulate and if vaccination campaigns are maintained and reinforced. Herd immunity occurs when a large part of a community becomes immune (through natural infection or vaccination) to a disease. As a result, the entire community is protected, not just those who are immune [26].

As of June 30, 2021, 13,917 confirmed cases of the COVID-19 have been reported in Togo. Extrapolating the prevalence of 65.5% to the 8 million Togolese, there would have been approximately 5.2 million cases of the COVID-19, corresponding to almost 375 times more than the number of cases notified in Togo at the same date. At the time of the study, only 3.1% of the population was vaccinated against COVID-19 [27]. This study confirms that cases are under-detected and under-notified. The under identification of cases is due to the particularity of this disease which is asymptomatic in more than 40.5% of the cases [28]. This clinical particularity makes it necessary to identify anti-SARS-CoV-2 antibodies through a serological survey in order to know the extent of the disease. Repeating such serological surveys, especially after the Omicron wave, would help refine the profile of the epidemic in the country. The under-reporting of cases is related to the low capacity of the epidemiological surveillance system to test and to take into consideration data coming from private and public health facilities. Indeed, a study in South Africa reported that more than 95% of the COVID-19 cases were not identified by the national surveillance system [29].

This is the first national seroprevalence study of SARS-CoV-2 in Togo and one of the few available in West Africa. The relatively large size of our sample allowed us to perform analyses according to vaccination status, sex, place of residence and age groups. We have thus partially filled the data gap and documented the actual level of SARS-CoV-2 circulation in the general population in Togo in 2021*.*

As in most cross-sectional studies, selection bias cannot be ruled out. Indeed, the districts concerned by the study were selected according to a reasoned approach on the basis of the incidence of the COVID-19 on March 1, 2021 at the time of the survey. In each health region, we included the districts with the highest and lowest incidence of the COVID-19. This choice meets WHO recommendations for SARS-CoV-2 sero-surveillance surveys [14]. Random selection of districts would have exposed us to the risk of including only districts with high incidence leading to an overestimation of SARS-CoV-2 seroprevalence or vice versa. In addition, to reduce the collinearity effect and avoid over representation of some households, we limited the number of subjects to be included in the households to 4.

In this study, a classification bias related to the use of ELISA tests was taken into account. No laboratory test is 100% sensitive and specific, and many likely have substantial measurement error. This measurement error could result in biased prevalence estimates [30]. To control for this type of bias, we adjusted the weighted seroprevalences using the sensitivity and specificity adjustment formula recommended by Sempos and Tian [30]. By stratifying on the COVID-19 vaccination status, we controlled for confounding bias in the multivariate analysis.

Finally, the results of the analysis of factors associated with the presence of anti-SARS-CoV-2 antibodies should be interpreted with caution. Indeed, in cross-sectional studies, it is difficult to establish the temporality between the exposure and the main event since both are collected at the same time.

## Conclusion

A household survey was conducted with the objective of estimating the seroprevalence of anti-SARS-CoV-2 antibodies in Togo at the national level in 2021. A total of 7593 subjects aged 5 years and older were enrolled. The overall weighted and adjusted seroprevalence was 65.5%. There were no differences by sex or age. In the unvaccinated subjects, neither residence nor health region was associated with the presence of antibodies. The results of this study show a high seroprevalence of SARS-CoV-2 infection and confirm the results observed in the West African region with nearly two thirds of the population having been in contact with the virus. Based on these findings, we make the recommendation to realise new seroprevalence studies coupled with antibody titration to confirm the dynamics of the epidemic and document the level of immunity in the population. This will help to guide the national vaccination policy against COVID-19.

## Data Availability

The datasets used and/or analysed during the current study are available from the corresponding author upon reasonable request.
